# Horses wait for more and better rewards in a delay of gratification paradigm

**DOI:** 10.3389/fpsyg.2022.954472

**Published:** 2022-07-22

**Authors:** Désirée Brucks, Anna Härterich, Uta König von Borstel

**Affiliations:** Animal Husbandry, Behaviour and Welfare Group, Institute of Animal Breeding and Genetics, University of Giessen, Giessen, Germany

**Keywords:** self-control, inhibitory control, delay of gratification, horses, coping behavior, error times

## Abstract

Self-control, defined as the ability to forgo immediate satisfaction in favor of better pay-offs in the future, has been extensively studied, revealing enormous variation between and within species. Horses are interesting in this regard because as a grazing species they are expected to show low self-control whereas its social complexity might be linked to high self-control abilities. Additionally, self-control may be a key factor in training and/or coping with potentially stressful husbandry conditions. We assessed horses’ self-control abilities in a simplified delay of gratification test that can be easily implemented in a farm setting. In Experiment 1, we gave horses (*N* = 52) the choice between an immediately available low-quality reward and a delayed high-quality reward that could only be obtained if the horse refrained from consuming the immediate reward. Different experimenters (*N* = 30) that underwent prior training in the procedures, tested horses in two test phases either with their eyes visible or invisible (sunglasses). Twenty horses waited up to the maximum delay stage of 60 s while all horses performed worse in the second test phase. In Experiment 2, we improved the test procedure (i.e., one experimenter, refined criterion for success), and tested 30 additional horses in a quality and quantity condition (one reward vs. delayed bigger reward). Two horses successfully waited for 60 s (quality: *N* = 1, quantity: *N* = 1). Horses tolerated higher delays, if they were first tested in the quantity condition. Furthermore, horses that were fed hay *ad libitum*, instead of in a restricted manner, reached higher delays. Coping behaviors (e.g., looking away, head movements, pawing, and increasing distance to reward) facilitated waiting success and horses were able to anticipate the upcoming delay duration as indicated by non-random distributions of giving-up times. We found no correlations between owner-assessed traits (e.g., trainability and patience) and individual performance in the test. These results suggest that horses are able to exert self-control in a delay of gratification paradigm similar to other domesticated species. Our simplified paradigm could be used to gather large scale data, e.g., to investigate the role of self-control in trainability or success in equestrian sports.

## Introduction

Being able to wait for something with a better outcome instead of going for an immediate but inferior outcome is advantageous in multiple situations (Beran, 2008). For example, while foraging it might pay to wait until sufficient gum has extruded from the tree before consuming it or to wait until the prey is in a favorable position before launching the attack (Stevens, 2014). But also, in social interactions it might prove beneficial for subordinate individuals to wait until the dominant ones have left the food resource before starting to feed (Johnson-Ulrich and Holekamp, 2020). The ability or capacity to invest more effort into obtaining a more valuable outcome instead of selecting a less valuable outcome has been termed self-control (see Beran, 2015 for a review). Self-control is essential in improving decision-making processes as it facilitates goal-directed behavior and ultimately future planning (Santos and Rosati, 2015). The ability of self-control is one aspect of inhibitory control, which additionally encompasses response or motor inhibition (regulation of impulsive motor actions) and cognitive inhibition (ability to control conditioned responses; Miller et al., 2019a). Self-control is generally assumed to be more cognitively demanding than response inhibition as it involves an additional decision component. Indeed, past research has shown that individual self-control abilities are linked to success in later life in humans (Shoda et al., 1990; Moffitt et al., 2011; but see Watts et al., 2018). Also, in chimpanzees, self-control is linked to other measures of general intelligence (Beran and Hopkins, 2018). This indicates that self-control plays an important role in cognitive processing of information.

While self-control is certainly beneficial in natural contexts [e.g., mate choice (Sozou and Seymour, 2003); foraging (Stevens and Stephens, 2010)], it might be equally important for domesticated animals that are no longer facing foraging decisions or mate choice but are subjected to various situations that most non-domesticated species would never experience in the wild. These situations include prolonged social isolation, frequently encountering unfamiliar individuals, living in barren and captive environments, and being subjected to procedures that might be stressful to the animals, to name just a few examples. In particular, farm animals are often kept in housing conditions that do not resemble their natural habitat (i.e., barren environments that limit expression of natural behaviors and induce frustration) and thus require flexible behavioral responses to cope with these conditions (e.g., Mason et al., 2013). Specifically, in these conditions, self-control is potentially beneficial as it allows individuals to flexibly adapt to their environment by optimizing decision-making processes in tempting and/or conflicting situations. It has been hypothesized that domesticated animals were selected for tamer and less aggressive behavior (Price, 1999), which might be linked to enhanced inhibitory control abilities in certain situations. Indeed, it has been shown that aggressiveness is linked to inhibitory control in a way that aggressive individuals often exhibit elevated impulsivity (e.g., hamsters: Cervantes and Delville, 2007; rats: Coppens et al., 2014; dogs: Gobbo and Zupan Šemrov, 2022). Furthermore, recent research found that inhibitory control is linked to emotional states in young chicken (Garnham et al., 2022). Accordingly, individual differences in inhibitory control might directly affect animal welfare, for example, explaining why some individuals cope with environmental or social conditions while others fail to do so. Individuals with better self-control might be less prone to develop stereotypical behaviors because they can restrain themselves from engaging in impulsive actions. Additionally, inhibitory control might play an important role during human-animal interactions. Animals with better inhibition might be less reactive in stressful situations and, thus, perceived as easier to handle. Self-control, in particular, might enhance attention in a training setting as the animals can better focus on the human signals instead of being tempted by the later reward.

Enormous variation in self-control abilities across animal species have been reported consistently. Accordingly, multiple hypotheses have been proposed as an attempt to explain the observed variation. These hypotheses are based on physiological explanations (Mayack and Naug, 2015; Miller et al., 2015), metabolic rate and longevity (Stevens and Mühlhoff, 2012; Stevens, 2014), brain size (MacLean et al., 2014) but also foraging ecology (Stevens et al., 2005) and social complexity (Amici et al., 2008; Lucon-Xiccato et al., 2022). However, considering that domesticated species show a decreased brain size (Kruska, 2007) and have an altered metabolic state depending on selection purposes (Rauw et al., 2017), some hypotheses might be difficult to test in domesticated species. Nevertheless, a handful of domesticated species have been tested in delay of gratification paradigms to assess their self-control abilities. On a group level, dogs waited between four to 25 times longer (Leonardi et al., 2012; Brucks et al., 2017b; Range et al., 2020) compared to pigs (Zebunke et al., 2018; Krause et al., 2021) and chicken (Abeyesinghe et al., 2005); however, domesticated animals generally exhibited rather low self-control abilities compared to other non-domesticated species (e.g., long-tailed macaques: Pelé et al., 2010; cleaner wrasse: Aellen et al., 2021). While certainly differences in experimental procedures and paradigms are accountable for some variation (Susini et al., 2021), more data on domesticated species’ self-control abilities, in particular of farm animals, is needed in order to better understand whether and how domestication affected self-control abilities.

In addition to this species-level variation, individual differences in inhibitory control abilities are frequently reported in studies. For example, sex (e.g., Brandão et al., 2019), age (e.g., Krause et al., 2021), food motivation (Meier et al., 2017; van Horik et al., 2017), social rank (Johnson-Ulrich and Holekamp, 2020), individual body conditions [i.e., hunger levels (Mayack and Naug, 2015)], but also the social environment (Lucon-Xiccato et al., 2022) can affect individual inhibition capacities. For example, chicken reared in a cognitively enriched environment exhibited poorer inhibitory control than chicken reared in a standard environment (Ryding et al., 2021). Consequently, to capture a species’ inhibitory control abilities, a large sample size is needed. Furthermore, it needs to be considered that not all measures of inhibitory control are necessarily tapping into the same behavioral construct (e.g., Bray et al., 2013; Marshall-Pescini et al., 2015; Brucks et al., 2017a); accordingly, comparative conclusions should only be drawn if either the same experimental paradigm is employed or if multiple different tests are used.

Horses (*Equus caballus*) have not been tested for their self-control abilities so far, even though they represent an interesting model species in this regard. Firstly, horses are generalist herbivores and can find food in rather evenly distributed patches (e.g., Salter and Hudson, 1979). This feeding ecology potentially requires only very little self-control as horses need to make only few decisions during foraging compared to carnivore or frugivore species that face resources with quickly changing availability and quality (Stevens et al., 2005). Nonetheless, horses show distinct resource preferences based on macronutrient and protein content (van den Berg et al., 2016) and sample from different foraging patches before making a choice (Devenport et al., 2005; Goodwin et al., 2007). Data on self-control in grazing species is missing so far. Secondly, horses live in complex social organizations that require high levels of social flexibility (Krueger, 2008; Maeda et al., 2021). According to the social complexity hypothesis, species living in complex social organizations that necessitate repeated interactions with various different partners possess enhanced inhibitory control abilities since they need to inhibit social behaviors in various situations (Amici et al., 2008; Johnson-Ulrich and Holekamp, 2020; but see Lucon-Xiccato et al., 2022). And thirdly, horses are domesticated species and high levels of inhibitory control are likely favorable for handling and training. In particular, self-control could be important in a training setting, as it might allow animals to better attend to human signals, and the prospect of appraisal (in case of correct responses), instead of focusing on immediate rewards. Whether domestication *per se* affected inhibitory control abilities (Marshall-Pescini et al., 2015; Gatto et al., 2018; Brucks et al., 2019), and self-control in particular (Range et al., 2020), is not clear; however, data of domesticated animals is particularly interesting for testing the links between self-control and animal welfare. As hypothesized also elsewhere (e.g., Langbein, 2018; Zebunke et al., 2018), individuals with better inhibition abilities might be better equipped for coping with stressful conditions, such as overcrowded housing, and lack of environmental stimulation. Especially horses are subjected to various housing conditions and training techniques that can affect trainability, handleability, and rideability (König von Borstel, 2013).

Various different experimental paradigms have been developed to test self-control abilities across animal species (see Miller et al., 2019a for a review). Broadly, these paradigms can be divided into two categories depending on the delivery mode of the rewards. In accumulation tasks, food items are delivered, either automatically via a remotely controlled device (Evans and Beran, 2007) or by an experimenter (Hillemann et al., 2014), one item at a time with a fixed interval between items until the subject starts to consume the accumulated food items. In exchange tasks, the subject is handed a less valuable reward and after a certain delay, this reward can be exchanged for a more valuable reward (e.g., Leonardi et al., 2012; Auersperg et al., 2013). Depending on the morphology of the animal species, this task is potentially inducing more or less temptation. For example, while a monkey can hold the food item with his/her hands, a dog would be required to hold the reward with his/her mouth and thus taste organ. Accordingly, the exchange task has been modified for some species to circumvent this potential confounding effect. For example, in dogs (Brucks et al., 2017b) and wolves (Range et al., 2020), rewards were delivered on retractable containers instead of handing the reward directly to the animals’ mouth. And recently, another experimental paradigm has been established, the so-called rotating tray task, in which the rewards are placed on a disk and rotate within reach of the animals without overt involvement of humans (e.g., Bramlett et al., 2012; Miller et al., 2019b; Brucks et al., 2021). While these paradigms certainly represent standardized tests for a laboratory setting, they are difficult to implement in an applied context as they require either larger apparatuses (e.g., rotating tray) or extensive training to familiarize the animals with the required action for obtaining the more valuable reward (e.g., exchange task).

In the current study, we aimed at establishing a simplified version of the exchange task that could be easily implemented in non-standardized environments (e.g., barns, stables, and meadows) and applied also by lay persons. Horses were given a choice between a less preferred immediate reward and a highly preferred but delayed reward presented on the experimenter’s hands in front of the horse. Considering that this experimental paradigm involves close and direct social interactions with an experimenter, the gaze of the experimenter could add an additional social inhibition component that could increase the horses’ success in the task. For example, dogs behave in a more inhibited way in a food context depending on whether the experimenter’s eyes are visible or not (Call et al., 2003). Also, horses are sensitive to human social cues (i.e., body orientation, gestures) (e.g., Proops and McComb, 2010) and potentially also gaze directions (Birke et al., 2011). Accordingly, we tested whether horses perform differently depending on whether the experimenters’ eyes are visible in Experiment 1. Based on the results of Experiment 1, we aimed at refining the protocol to allow a better comparison of horses’ self-control abilities with other species. Consequently, we adopted similar procedures as used in other studies, in terms of reward types, delay stages, criteria for success, and also tested horses in a more standardized setting. In Experiment 2, we tested a new population of horses in two conditions, a quality (less preferred reward vs. highly preferred reward) and a quantity (one reward vs. multiple reward items) condition. To find out whether certain behaviors facilitated waiting success, we coded the horses’ behavior during the test. Furthermore, to explore whether individual horse characteristics (i.e., nervousness, trainability, patience, and food motivation) are linked to self-control and whether horse owners can assess their horses’ self-control, we asked the horse owners to fill in a questionnaire.

Horses are a grazing species and thus potentially require little self-control during foraging but also live in a complex social environment that potentially requires enhanced self-control abilities. Accordingly, two mutually exclusive hypotheses can be derived: if horses show good self-control abilities this could be seen as support for the social complexity hypothesis; however, a lack of self-control would support the feeding ecology hypothesis. Furthermore, we hypothesize that distraction behaviors emitted during the waiting period facilitate waiting success. Accordingly, horses that show more distraction behaviors are expected to be more successful in delaying gratification than horses showing fewer of these distraction behaviors.

## Experiment 1

### Methods

#### Subjects and housing

We tested 56 privately-owned horses of various breeds in a delay of gratification paradigm. Four horses did not complete testing due to health-related issues (*N* = 2) and frustration/aggression during the test (*N* = 2), one horse developed aggressive behaviors during the course of the second phase of the test, accordingly, data from the first test phase could still be collected from this horse. Thus, in total, 52 horses (29 F/23 M; age: 15.1 ± 7.0 years, range: 3.5–30.5 years) of various breeds (see Supplementary Data 1 for individual characteristics) were included in the analyses. The horses were kept in group-housing conditions (*N* = 32) or in individual boxes with daily access to outdoor areas (*N* = 20). Hay was provided either *ad libitum* (*N* = 20) or in a restricted manner (3–5× a day; *N* = 32).

The tests were conducted by thirty different experimenters (3 M/27 F) that were familiar with the horses (e.g., owner of horse, rider of horse, horse from same social group as own horse). Each experimenter tested two horses. Due to the exclusion of four horses, data from four experimenters that tested only one horse were present in the data set. To ensure reliability between the different experimenters, they were required to complete a training session at the beginning of data collection. This training session included reading the detailed procedures and providing a video sequence of performing the food preference test as well as the training phase (see description below). The study coordinator (DB) checked all videos and gave feedback in case that the procedure was not performed correctly. Only when the procedures were applied accurately, the experimenters were allowed to start data collection.

#### Experimental procedures

Each horse was tested individually in a box or paddock. The horses were either free to move or tied to the wall in case no box was available. In case that a door from a box or a stranded wire (not electrified) was used to separate the horse and experimenter it was ensured that the horses could easily reach across the barrier with their head and neck. The test areas were chosen to minimize distractions during the test. The experimenters stood in front of the horse at a distance of 1–2 m (depending on the horse’s size) and were instructed to behave passively during the test (i.e., no verbal commands or gestures).

The food rewards were stored on the ground behind the experimenter and out of reach for the horses. Instead of presenting the food items on containers or a rotating tray as in previous studies, we presented the food items on the experimenter’s hands in front of the horse but out of reach (similar to e.g., Leonardi et al., 2012; Auersperg et al., 2013). Food was presented on open hand palms on each side of the body (approximately 40 cm distance between hands) at the height of the experimenter’s hip (see Supplementary Video). The distance between both hands and the horse’s head was between 20 and 30 cm at the beginning of each trial (‘start position’). This distance was maintained in a dynamical way, i.e., if a horse reached forward with extended head, the experimenter could take a step back to avoid that the horses reached the delayed food reward before the respective delay-time was over.

#### Food preference test

To find a highly preferred food reward and a less preferred but still consumable food reward for each horse, we conducted a food preference test at the beginning of the experiment. Horse owners were asked about their horses’ preferences and to validate these suggestions, the horses were repeatedly offered both reward types simultaneously. To ensure that the horses would consume the less preferred reward consistently, if no better reward was offered, the horses were presented with one piece of the owner-suggested low-value reward (LVR) at a time. This was repeated for a total of 10 trials. If the horse readily consumed each piece of the reward type, it was used as LVR in the subsequent test. For all of the horses in Experiment 1, hay (either as a loose bundle or as cobs) was used as LVR.

Once the LVR was determined, the horses were presented with a choice between the LVR and a high value reward (HVR; e.g., carrot, apple, and banana; see Supplementary Material for HVRs per horse). The experimenter visibly placed one piece of the HVR on one hand and a small bundle of hay (=LVR) on the other hand. Both hands (with open palms) were presented to the horse for 3 s before both hands were simultaneously stretched forward within the horses’ reach. The horses were allowed to select and consume one of the rewards while the experimenter retracted the hand holding the food reward that was not selected (see Supplementary Video). The horses’ choice was noted and the next trial was started. To prevent horses from developing a side preference, the sides of the HVR and LVR were alternated between each trial.

Twenty trials were conducted per session. If a horse selected the HVR in 16 out of the 20 trials (one-sided binomial test: *p* = 0.001), he/she reached the criterion and the HVR was used in the subsequent test. In case that a horse did not reach the criterion, the food preference test was repeated for a total of three sessions. If no preference was shown, a different reward type was used as HVR. If a horse did not reach the criterion within six sessions in total, she/he was excluded from the study. Some horses developed side preferences and the experimenter performed 15 trials with food only on the non-preferred hand to counteract this preference. Following this step, another food preference session was conducted.

#### Training

In the training phase, the horses were familiarized with the test procedure and the concept of gaining access to the HVR only if the LVR is not consumed. For each horse, it was randomly determined on which hand the less preferred and highly-preferred reward was positioned and these sides were kept constant throughout the study. To ensure that the horses were paying attention to the test, the experimenter initiated a trial only if the horse was looking toward the experimenter. If a horse was not attentive (e.g., head turned away), the experimenter called the horse by her/his name and made an attention-getting sound that the horse was familiar with (e.g., clicking with tongue or whistling). In case that this was not successful, the same procedure was repeated twice; however, if a horse was still not attentive, the test was terminated and repeated at a later point in time.

At the beginning of each trial, both reward types were presented on the hand palms for 3 s in the start position. The hand holding the LVR was now stretched out within reach of the horse while the hand holding the HVR remained in the start position out of reach (see Supplementary Video). If the horse did not consume the LVR, the hand with the HVR was also stretched forward after 1 s had passed. If the horse consumed the LVR, the hand holding the HVR was instantly closed and withdrawn. In total, 15 trials were conducted per session. Horses reached training criterion, if they refrained from eating the LVR and instead waited for the HVR in at least twelve of the 15 trials (one-sided binomial test: *p* = 0.004). If this criterion was not reached, another session was conducted. In case of horses not reaching this criterion within six sessions they were excluded from the experiment.

#### Test phase

Horses that reached the training criterion entered the test phase, in which the delay between the immediately available LVR and the delayed HVR was increased in a stepwise manner depending on each horse’s success. As before, both reward types were presented simultaneously at the beginning of each trial on the predetermined hands (‘start position’) for 3 s before stretching the hand holding the LVR within the horse’s reach while the hand holding the HVR remained in the start position and hence out of reach (see Supplementary Video). Both hands remained motionless throughout the trial duration. If the horse did not consume the LVR, the HVR was made available by stretching the hand forward after the delay had passed. If a horse consumed the LVR, the hand holding the HVR was closed and withdrawn. The next trial started after an inter-trial interval of 5–10 s, once the horse had finished chewing.

Per session 15 trials were conducted. Up to three sessions were performed per day with at least a 5-min break in-between sessions (see Table 1 for overview). Each test was video recorded. If a horse waited for the HVR in at least three out of 15 trials within a session, he/she proceeded to the next delay stage. The delay between LVR and HVR was increased in a stepwise manner depending on the horses’ success, starting at 2 s, then 5, 10, 15, 20, 25, 30, 40, 50, and up to a maximum of 60 s. If a horse did not reach this criterion, the session was repeated. A maximum of six sessions was conducted per delay stage and if a horse did not reach the criterion within these six sessions, the test was terminated.

**TABLE 1:** Overview and order of training and test procedure (for a horse assigned to the eyes visible-first test group).

Order	Phase	Choice	Delay	Sessions	Trials	Criteria
1	*Food Preference Test*	LVR vs. HVR	–	Until criterion reached or max. 6	20	Choice for HVR in at least 16 trials
2	*Training*	LVR vs. HVR after 1 s	1 s	Until criterion reached or max. 6	15	Waiting for HVR in at least 12 trials
3	*Test – eyes visible* ^a^	LVR vs. HVR after Xs	2, 5, 10, 15, 20, 25, 30, 40, 50, and 60 s	Until criterion reached or max. 6 per delay stage	15	Waiting for HVR in at least 3 trials
4	*Test – eyes invisible* ^a^	LVR vs. HVR after Xs	2, 5, 10, 15, 20, 25, 30, 40, 50, and 60 s	Until criterion reached or max. 6 per delay stage	15	Waiting for HVR in at least 3 trials

^a^The order of the two test phases was counterbalanced across horses. Once a horse had reached her/his max. delay stage, the next phase started at the 2 s delay.

The horses were tested until they gave up waiting or reached the maximum delay stage of 60 s. All horses were tested in two test phases: (1) *eyes visible*: the experimenter directly gazed at the horse during the whole delay duration; (2) *eyes invisible*: the experimenter wore sunglasses during the test, thus, shielding the eyes. Horses were randomly assigned to start with either of the two test phases (eyes visible first: *N* = 25; eyes invisible first: *N* = 31) and switched to the respective second phase (at the 2 s delay) once they gave up waiting or once they had reached the maximal delay stage of 60 s.

### Analyses

For each test session, the number of choices for the HVR (=waiting) as well as number of trials waiting for the LVR (=not waiting) was noted. Based on this data the maximum delay stage (last delay stage that was successfully completed) was extracted for each horse. If a horse did not pass the training criterion, it was excluded from the analyses (*N* = 4). If a horse did not pass the 2 s delay stage in the test phase, a maximum delay of 0 s was entered. If a horse successfully passed all delay stages up to the maximum delay stage, 60 s was entered as maximum delay.

A second coder coded 15% of the videos. To assess inter-observer reliability, we calculated the intra-class correlation coefficient (ICC) using the ‘irr’ package (version 0.84.1; Gamer et al., 2019). Consistency between coders was very good [ICC (two-way, consistency): LVR choices: ICC = 0.949, *p* < 0.001].

To assess whether individual and environmental factors affect the maximum delay stage tolerated by horses, we fitted an ordinal mixed model [i.e., cumulative linear mixed model (CLMM) with a logit-link function (Agresti, 2002)]. Maximum delay (factor: 0, 2, 5, 10, 15, 20, 25, 30, 40, 50, and 60) was set as the response variable while age in months (numeric), sex (factor: F, M), test phase (factor: eyes invisible and visible), test order (numeric: 1 and 2), housing (factor: group and individual), and roughage feeding management (factor: *ad libitum* and restricted) were included as predictors. An interaction term between test phase and test order was included to assess whether starting with a particular test phase influenced the maximally tolerated delay time. To control for repeated testing of horses and the involvement of different experimenters, we included horse ID and experimenter ID as random effects.

As a means to avoid ‘cryptic multiple testing’ (Schielzeth and Forstmeier, 2009), the full model was compared to a conceptual null model (comprising only housing and sex as predictors). Significance was determined by dropping one predictor at a time from the full model and compared the results with the full model using the *drop1* function. Model comparison was done by utilizing a likelihood ratio test (Dobson and Barnett, 2018). Considering that each horse was tested only twice (i.e., once in each test phase), random slopes were not identifiable. Prior to fitting the model, we checked the distribution of age, which was symmetrical, and subsequently scaled age to a mean of 0 and a standard deviation of 1 to facilitate model convergence.

The model was fitted using R (version 4.0.2; R Core Team, 2021) using the function *clmm* of the package ‘ordinal’ (version 2019.12-10; Christensen, 2019). Model assumptions of the CLMM (i.e., proportional odds, collinearity, and model stability) were assessed (see Supplementary File for diagnostics). Confidence intervals (95%) of the estimates and fitted values were determined by a parametric bootstrap (*N* = 1000 bootstraps) using a function kindly provided by Roger Mundry. The data set included 95 observations from 48 horses (note that horses that failed the training criterion were excluded from the analyses).

### Results

**FIGURE 1:**
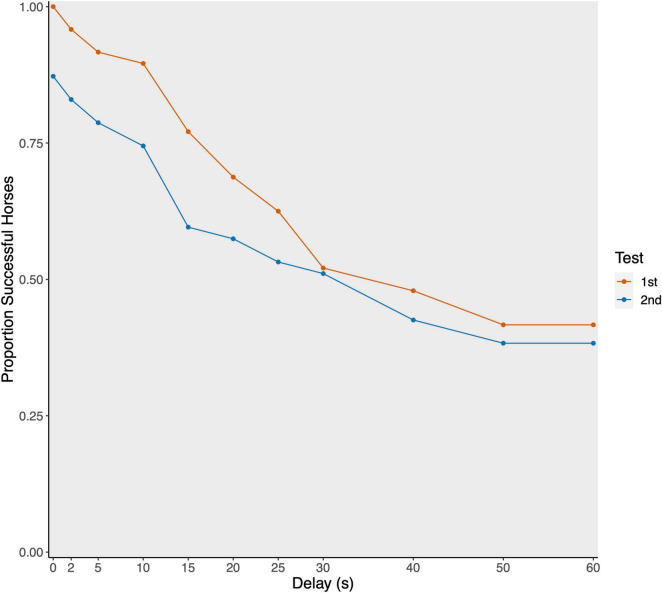
Proportion of successful horses per delay stage plotted separately for first (red; *N* = 52) and second (blue; *N* = 48) test.

On a group level, the horses tolerated a maximum delay of 36.08 ± 22.85 s (mean ± SD; median: 40 s). Twenty horses (41.67%) reached the maximum delay stage of 60 s (see Figure 1).

The full-null model comparison revealed that the full model described the data significantly better than the conceptional null model (Likelihood Ratio Test: χ^2^ = 16.02, df = 5, *p* = 0.007).

**FIGURE 2:**
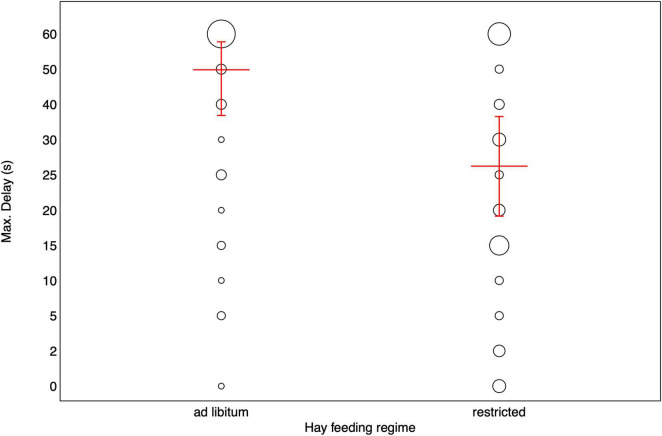
Maximum delay stages reached by horses fed hay *ad libitum* and restricted. Bubbles depict the frequency of maximally tolerated delay stages while the size of the bubbles corresponds to the number of horses [range: 1 (smallest bubble) – 23(largest bubble)]. The red horizontal bar depicts the fitted model and the error bars show the confidence limits for all other variables in the model centered to a mean of zero.

The interaction between test phase and order did not reveal significance (see Table 1). Likewise, no effect of sex or housing on the maximally tolerated delay stage could be detected. Older horses tended to reach higher delay stages compared to younger horses. And horses that had access to hay *ad libitum* tolerated higher delays (45.63 ± 20.01 s) compared to horses fed hay in a restricted manner (29.15 ± 22.43 s; see Table 2 and Figure 2). Since the interaction between test phase and order was not significant, we removed the interaction from the model to assess the main effects of the two variables (see Supplementary Table 3 for the estimates of the reduced model). Accordingly, test phase (eyes visible, invisible) did not significantly affect the horses’ performance (CLMM: −0.186 ± 0.436, *p* = 0.669), however, the horses waited only for shorter delays in the second test (33.60 ± 24.47 s) compared to the first test (38.52 ± 21.12 s; CLMM: −0.931 ± 0.459, *p* = 0.038; Figure 1).

**TABLE 2:** Effects of predictors on maximum delay duration based on CLMM with horse and experimenter as random effects (full model).

Term	Estimate	*SE*	lower CI	upper CI	Chisq	df	*P*-value
0| 2	−6.409	1.888	−11.599	−3.187			^2^
2| 5	−5.663	1.837	−10.499	−2.378			^2^
5| 10	−5.092	1.800	−9.453	−1.826			^2^
10| 15	−4.719	1.775	−9.145	−1.521			^2^
15| 20	−3.383	1.692	−7.530	−0.266			^2^
20| 25	−2.933	1.669	−6.909	0.208			^2^
25| 30	−2.509	1.650	−6.623	0.578			^2^
30| 40	−2.025	1.637	−6.032	1.097			^2^
40| 50	−1.520	1.630	−5.484	1.614			^2^
50| 60	−1.052	1.626	−4.771	2.133			^2^
Phase (eyes invisible)	1.309	2.558	−3.854	6.472			^2^
Order	−0.443	0.930	−2.480	1.326			^2^
Age^1^	0.846	0.477	−0.026	1.902	3.242	1	0.072
Sex (male)	0.648	0.820	−0.956	2.317	0.643	1	0.422
Feeding (restricted)	−2.239	0.873	−4.555	−0.733	7.391	1	**0.007**
Housing (individual)	0.094	0.886	−1.657	1.970	0.011	1	0.916
Phase (eyes invisible) × order	−1.006	1.697	−4.406	2.359	0.363	1	0.547

^1^Age was *z*-transformed to a mean of zero and standard deviation of one. Original variable: 176.71 ± 84.60 months.

^2^Not shown due to limited interpretability. Significant effects (p < 0.05) are highlighted in bold.

## Experiment 2

### Methods

#### Subjects and housing

We tested 30 horses [19 M/11 F; age (mean ± *SD*): 16.1 ± 6.2 years, range: 5.4–27.8 years; see Supplementary Data 1 for details] of different breeds. Some horses were privately-owned (*N* = 14) while other horses belonged to an equestrian center (*N* = 16). The horses were kept either in individual boxes with daily access to outdoor areas (*N* = 15) or in group-housing conditions (*N* = 15). To ensure an equal food motivation between horses that were fed hay *ad libitum* and horses that were fed hay in a restricted manner 2–3 times a day, all tests were conducted 0.5 h after the horses consumed their hay portion (restricted feeding) or at times of the day when the horses had not fed from the hay for at least 0.5 h (*ad libitum* feeding).

Owners were asked to fill in a questionnaire prior to starting the data collection. The questionnaire included questions related to (1) the horses’ general trainability (How easily does your horse learn novel skills? How would you rate your horse’s trainability?), (2) food motivation (How insistent is your horse when you have food in your bag? How food motivated is your horse?), and (3) coping abilities (How skittish is your horse? How patient is your horse? How susceptible to stress is your horse?). The questions could be answered on a 6-point Likert scale (see Supplementary Data 3 for details).

#### Experimental procedure

The experimental setup and procedures were identical to those employed in Experiment 1; however, we made some small adjustments: All horses were tested individually in the same test-box (approx. 3 m × 3 m) with a nylon stable guard fixed to the door adjusted to chest height to ensure that all horses could reach across the box door independent of their height. To avoid visual distractions during the test, a fabric panel construction (1.85 m height) was set up around the test arena (see Supplementary Video).

The horses were tested in two conditions: a *quality condition* with the choice between an immediately available low-value reward (LVR) and a delayed high-value reward (HVR). And a *quantity condition*, in which the horses were given the choice between an immediately available low quantity reward (LQR; 1 piece of reward) and a delayed high quantity reward (HQR; 5 pieces of reward). These quantity differences were selected as horses have been shown to successfully discriminate between even smaller quantities (Uller and Lewis, 2009; Petrazzini, 2014) (but see Henselek et al., 2012). Half of the trials in the quantity condition comprised the LVR reward (i.e., 1 vs. 5 pieces of LVR) and half of the trials the HVR (1 vs. 5 pieces of HVR). The order of LVR and HVR trials was semi-randomized with each trial type no more than twice in a row. Accordingly, two food preference tests were conducted, one for the quality condition and a separate preference test for the quantity condition [using the same criterion as in Experiment 1 (i.e., 16 out of 20 trials choice for HQR/HVR)] prior to starting with the respective condition (see Supplementary Table 4 for timeline). Likewise, the training phase was conducted twice (once for each test condition). The horses were randomly assigned to start either with the quality condition (*N* = 15) or the quantity condition (*N* = 15) before switching to the respective other once they stopped waiting. In-between the two test conditions a break of 2 weeks was implemented.

And, horses were tested until they gave up waiting (i.e., not reaching criterion within 5 sessions) with no fixed upper delay limit. The order in which horses were tested was randomized per day and counterbalanced in total in a way that each horse was at least once tested first or last. Test sessions were performed on four consecutive days followed by a 3-day break. One female experimenter (AH) tested all horses wearing sunglasses throughout the test to minimize provision of subconscious mimic-based cues to the horse.

### Analyses

#### Behavioral coding

The videos were coded using Solomon Coder (2015 by András Péter). The subject’s choice (HVR or LVR) was coded as well as the latency to consume the reward. Furthermore, we analyzed the horses’ behavior during the delay duration. Specifically, we coded the distance to the hand holding the LVR, the horses’ attention, and other behaviors, such as oral manipulations, head movements, chewing, Flehmen, pawing, as well as reward-directed behaviors (see Table 3 for detailed descriptions).

**TABLE 3:** Ethogram of coded behaviors.

Category	Variable	Description
Latency choice	Latency to consume LVR/HVR	Time from start of trial until horse either closed his/her lips around the LVR or until the delay time has passed
Attention	Look away	Head turned away in >45° angle from experimenter
Distance	Large distance	Horses’ mouth is more than 0.5 m away from the hand holding the LVR
Other behaviors	Empty chew^a^	Horse chews without having food in his/her mouth
	Oral manipulation	Licking, nibbling or biting into barrier/box/door or own body parts
	Head movement	Any repeated movements with the head (i.e., horizontal and vertical movements or rotational movements)
	Flehmen	Lifting the upper lip, usually associated with a forward stretched neck
	Pawing	Repeatedly lifting one leg and scratching with the hoof on the ground
Reward-directed behaviors	Sniffing LVR	Sniffing on LVR without taking it into the mouth
	Pushing LVR	Pushing hand holding LVR away with mouth or head

^a^Only coded after 10 s of a trial had elapsed to avoid coding instances of horses still chewing the previous reward.

A second coder coded 10% of the videos and inter-observer reliability was calculated. Consistency between coders was good [ICC (two-way, consistency): LQR choices: ICC = 0.992, *p* < 0.001; looking away: ICC = 0.991, *p* < 0.001; head movements: ICC = 0.719, *p* < 0.001; large distance to food: ICC = 0.953, *p* < 0.001; pawing: ICC = 0.988, *p* < 0.001; chewing: ICC = 0.959, *p* < 0.001; sniffing LVR: ICC = 0.912, *p* < 0.001; pushing LVR: ICC = 0.810, *p* < 0.001; latencies to take food: all ICC > 0.842, all *p* < 0.001].

#### Statistical analyses

For each horse, the maximally tolerated delay stage was extracted. Furthermore, the behaviors recorded during the waiting duration (i.e., large distance, looking away, chew, oral manipulation, head movement, flehmen, and pawing) were summed up and subsequently divided by the total session duration to account for differences in length between sessions. Reward-directed behaviors were not included in the analysis, as these were observed only in a subset of horses (*N* = 6) and occurred rarely (median ± SD: 0.00 ± 0.08 proportion per test duration).

Similar to the analyses of Experiment 1, we wanted to find out whether individual characteristics affected individual performance in the test; accordingly, we included age, sex (factor: female and male), as well as housing (factor: individual and group) and feeding management (factor: restricted and *ad libitum*) as predictors. Furthermore, to assess whether horses performed better in the quality or quantity condition and whether the order of these tests matters, we included an interaction term between test condition (factor: quality and quantity) and test order (numeric: 1 and 2) into the model. To account for repeated testing of horses, horse ID was included as a random effect. Considering that each horse could reach only two maximum delay stages, no random slopes were identifiable. Prior to fitting the model, we *z*-transformed age to a mean of 0 and a standard deviation of 1.

Initially, we aimed at fitting a cumulative linear mixed model (CLMM) to model maximum delay as a factor; however, the proportional odds were strongly violated (see Supplementary Table 6). Consequently, we switched to fitting a generalized linear mixed model (GLMM1) with a poisson error distribution and a log-link function using the *glmer* function within the ‘lme4’ package (version 1.1–27.1; Bates et al., 2015). The same predictors were entered but maximum delay was included as a numeric variable. Model assumptions were checked prior to fitting the model (i.e., no zero-inflation was detectable and residuals of the random intercepts were symmetrical; see Supplementary File for additional diagnostics). We obtained confidence intervals via the *bootMer* function (*N* = 1000 bootstraps) within the lme4 package. The data set used for the maximum delay analyses consisted of 49 observations from 29 horses.

For ruling out that horses’ performance was affected by satiation due to the high number of trials per session, we ran an additional binomial model (GLMM2) with success (binary: waiting/not waiting) as response variable and trial number (numeric: 1–15) as predictor. Horse ID was entered as random effect and trial number as random slope (see Supplementary File for details).

Verifying that the owners’ answers to the questionnaire were indeed tapping into the same behavioral construct as intended, we ran correlations between all seven questions (see Supplementary Data 3). Variables that exhibited a high correlation, were averaged for further analyses. For assessing whether the owners’ assessment of their horses’ self-control abilities, general trainability, food motivation, coping abilities, and patience were linked to the individually reached maximum delay times, we ran Spearman correlations with a Bonferroni correction for multiple testing. For this analysis, we used only the highest delay stage that each horse reached in the test.

To analyze how coping behaviors affected individual waiting success within a session, we fitted a logistic generalized linear mixed model (GLMM3). The response variable was entered as a two-column matrix with the number of successes (=choice HVR) and the number of failures (=choice LVR) per individual using the *cbind* function (Baayen, 2008). Since we were interested in the influence of coping behaviors as delays increased and whether horses used these coping behaviors differently across the two test conditions, we included two interaction terms between coping and delay, and between coping and test phase into the model. Furthermore, age and sex were included into the model as control variables. As random effects, we included horse ID and session number nested within horse ID.

To avoid overconfident model estimates and to maintain the type I error rate at 0.05, random slopes were included into the model (Schielzeth and Forstmeier, 2009). Delay (as a numeric variable) and coping behaviors were included as slopes for the intercept of session number nested in horse ID; and delay (as numeric variable) as well as an interaction between test phase (manually dummy coded and centered to a mean of 0) and coping behaviors were included as slopes for the intercept of horse ID.

The model was fitted using the *glmer* function with the ‘lme4’ package (version 1.1–27.1; Bates et al., 2015) with a binomial error distribution and logit-link function. After an exploratory analysis, we decided to set the 10 s delay stage as reference level for the delay variable as this seems to be a more biologically relevant stage than the 2 s delay (see Supplementary File for details). Confidence intervals were obtained via parametric bootstrapping (*N* = 1000 bootstraps) using the function *bootMer* within the lme4 package. The data set for this analysis consisted of 551 observations form 29 horses.

To assess whether the horses’ success in the quantity condition differed between trials with the HVR and the LVR, we fitted an additional binomial GLMM (GLMM4) with the number of waiting success (=choice delayed option) and the number of failures (=choice immediate option) as a response matrix. As predictors, we included age (*z*-transformed to mean of 0 and standard deviation of 1), sex (factor: female and male) and an interaction term between reward type (factor: LVR and HVR) and delay stage (factor: 0, 2, 5, 10, 20, 30, 40, 60, and 80 s). To account for repeated testing of horses, we included two random intercept terms: horse ID and session number nested within horse ID. Delay (numeric) and reward type (factor) were included as random slopes for both random effects terms (see Supplementary File for model diagnostics).

Deciding early on in a trial whether it is worth waiting or not is generally seen as an indication for anticipation of the upcoming delay duration, as it does not pay to invest energy into a resource that is devaluated by a large delay (e.g., Pelé et al., 2010; Auersperg et al., 2013). To find out whether the horses gave up waiting at a random time point within each trial or rather at the beginning or end of a trial, we ran an analysis of the error times. The distribution of observed error times (=latency to consume LVR) was compared with the distribution of error times expected under the null hypothesis of a constant giving up chance during the trial. If horses can anticipate the upcoming delay duration, we would expect that horses decide early on in a trial whether the delayed reward is worth waiting for. Using a Kaplan–Meier survival analysis, we calculated the estimated probability to continue waiting at each time point that the horses gave up waiting. The error times (i.e., time point at which the LVR was consumed) as well as successful trials (i.e., successfully waiting for HVR) were entered as censored data. The survival probability (chance to wait longer than elapsed time within a trial) and the expected distribution (chance to wait under null hypothesis) were compared using an adjusted Kolmogorov–Smirnov test (Haccou and Meelis, 1992). The analysis was run only for the delay stages above 5 s.

### Results

#### Maximum delay

In the second experiment, seven horses did not pass training in the quality condition (25%) while all horses passed the training in the quantity condition. One horse was not food motivated and was excluded at the beginning of the study. An additional horse lost interest in the test and refused to participate at some point during the second test condition (the first test condition was still included in the analyses) and another horse did not pass the food preference test for the quality condition.

**FIGURE 3:**
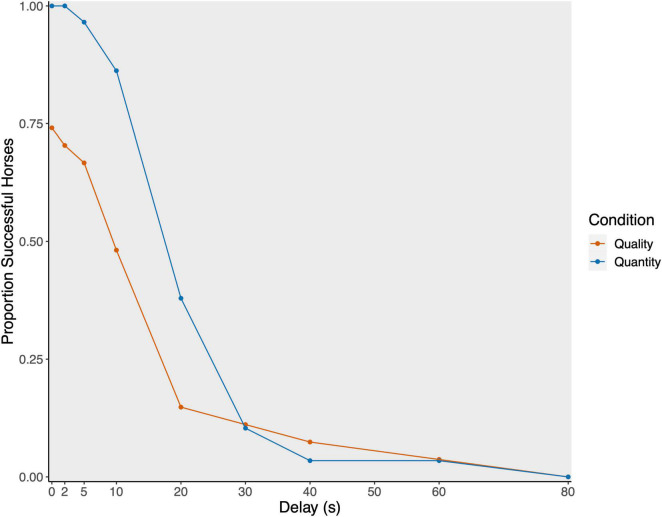
Proportion of successful horses per delay stage plotted separately for quality (red; *N* = 28) and quantity condition (blue; *N* = 29).

On a group level, the horses tolerated a delay of 13.35 ± 14.45 s (median: 10 s) in the quality and 15.07 ± 11.17 s (median: 10 s) in the quantity condition (see Figure 3).

**FIGURE 4:**
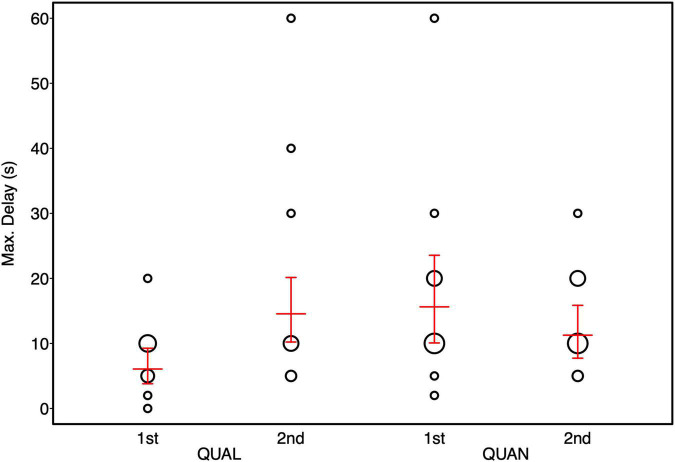
Maximum delay stages reached by horses in the quality (QUAL) and quantity (QUAN) test condition as a function of test order. Bubbles depict the frequency of maximally tolerated delay stages while the area of the bubbles corresponds to the number of horses (range: 1–7). The red horizontal bar depicts the fitted model and the error bars show the confidence limits for feeding management centered to a mean of zero.

Overall, the full model fitted the data significantly better than the null model (Likelihood ratio test: χ^2^ = 11.11, *p* = 0.049). Age did not significantly affect individual performance (see Table 4). Horses fed hay *ad libitum* tended to reach higher delay stages than horses fed hay in a restricted manner. Furthermore, we could detect a significant interaction between condition and test order. Horses that started with the quantity condition performed better in the quality condition compared to horses that started with the quality condition first (see Table 4 and Figure 4).

**TABLE 4:** Effects of condition, order, age, and feeding management on maximally reached delay durations (full model; GLMM1).

Term	Estimate	*SE*	Lower CI	Upper CI	Chisq	df	*P*-value
Intercept	0.980	0.484	−0.006	1.947			^2^
Condition (QUAN)	2.077	0.662	0.689	3.403			^2^
Order	0.946	0.314	0.296	1.578			^2^
Age^1^	0.073	0.096	−0.127	0.257	0.557	1	0.456
Feeding (restricted)	−0.431	0.227	−0.859	0.031	3.403	1	0.065
Condition (QUAN) × Order	−1.202	0.421	−1.994	−0.318	7.382	1	**0.007**

^1^Age was *z*-transformed to a mean of zero and standard deviation of one. Original variable: 197.06 ± 77.74 months.

^2^Not shown due to limited interpretability. Significant effects (p < 0.05) are highlighted in bold.

We found no effect of trial number on success (GLMM: −0.008 ± 0.007, *z*-value = −1.146, *p* = 0.252), accordingly, horses were equally likely to wait in the beginning, middle, and end of a session (see Supplementary File for details).

The owners’ assessment of their horses’ self-control abilities was not related to the maximum delay stage that their horse reached (Spearman: *N* = 29, *r*_s_ = 0.02, *p* = 0.903). Furthermore, none of the other variables showed a significant correlation with the horses’ performance in the test (see Supplementary Data 3).

#### Influence of distraction behaviors on waiting performance

The full model fitted the data significantly better than the conceptual null model with only sex and age as predictors (Likelihood ratio test: χ^2^ = 130.42, df = 17, *p* < 0.001). Horses that showed more coping behaviors were more successful, in particular during the delay stages of 20, 30, and 40 s compared to the 10 s delay stage (see Table 5 and Supplementary Figure 2). During lower delay stages (i.e., 2 and 5 s), coping behaviors did not affect waiting success. For the high delay stages (60 and 80 s), coping behaviors were not linked to waiting success either. Coping behaviors did not differ between the two test conditions (see Table 5).

**TABLE 5:** Effects of age, sex, amount of coping behaviors, test phase and delay on number of successful trials (GLMM3).

Term	Estimate	*SE*	Lower CI	Upper CI	Chisq	df	*P*-value
Intercept	−0.709	0.640	−1.894	0.518			^2^
Age^1^	0.130	0.390	−0.613	0.956	0.110	1	0.740
Sex (M)	0.054	0.777	−1.402	1.442	0.005	1	0.945
Coping behav^1^	1.304	0.288	0.727	1.980			^2^
Phase (Quan)	0.652	0.382	−0.080	1.441			^2^
Delay 2 s	2.361	0.29	1.805	2.897			^2^
Delay 5 s	1.462	0.194	1.101	1.842			^2^
Delay 20 s	−4.165	0.352	−4.886	−3.537			^2^
Delay 30 s	−7.874	0.718	−9.428	−6.656			^2^
Delay 40 s	−12.548	1.372	−13.937	−11.037			^2^
Delay 60 s	−15.747	3.461	−16.692	−14.395			^2^
Delay 80 s	−26.931	4.613	−27.802	−25.980			^2^
Cope:Phase	−0.314	0.316	−0.955	0.370	0.972	1	0.324
Cope:Delay 2 s	−0.023	0.212	−0.582	0.526	^3^	^3^	0.914
Cope:Delay 5 s	0.229	0.173	−0.191	0.645	^3^	^3^	0.185
Cope:Delay 20 s	0.553	0.185	0.026	1.084	^3^	^3^	**0.003**
Cope:Delay 30 s	0.864	0.243	0.138	1.734	^3^	^3^	**<0.001**
Cope:Delay 40 s	1.341	0.468	0.147	2.474	^3^	^3^	**0.004**
Cope:Delay 60 s	0.314	1.188	−0.963	1.539	^3^	^3^	0.791
Cope:Delay 80 s	1.924	1.367	0.920	3.262	^3^	^3^	0.159

Note that the 10 s delay stage was set as reference level for the delay variable.

^1^Variables were scaled to a mean of 0 and a standard deviation of one. Original variables (mean ± SD): age = 197.06 ± 77.74 months; proportion of coping behaviors per test duration = 0.40 ± 0.38.

^2^Not depicted due to limited interpretability.

^3^Likelihood ratio test for coping × delay: Chisq = 20.296, df = 7, *p* = 0.005. Significant effects (p < 0.05) are highlighted in bold.

In the quantity condition, the full model containing the reward type – delay stage interaction did not explain the data better compared to the null model lacking the reward type term (LRT: χ^2^ = 9.05, df = 8, *p* = 0.338). Accordingly, the interaction between reward type and delay was not significant (LRT: χ^2^ = 3.77, df = 7, *p* = 0.805). To assess whether reward type affected waiting success as a main effect, we fitted a reduced model lacking the interaction term between reward type and delay stage; however, also as a main effect, reward type did not significantly affect waiting success (GLMM: −0.035 ± 0.202, *z* = −0.174, *p* = 0.862; see Supplementary Table 11 for complete model results).

#### Error times

The majority of horses (21 out of 29 horses) gave up waiting at the beginning of trials instead of at random time points throughout the trials (see Supplementary Data 2). These early error times occurred more often during higher delay times than during lower times. Interestingly, the majority of horses gave up waiting earlier than expected by a constant giving up chance in the quality condition (i.e., in higher delay stages; 75% of horses) while only half of the horses gave up waiting significantly earlier than expected in the quantity condition (see Supplementary Material).

## Discussion

We found that horses were able to wait for a delayed reward of better quality and quantity up to 60 s in a delay of gratification paradigm. Individual variation in self-control was consistently explained by hay feeding management in both experiments as horses having access to hay *ad libitum* reached higher delay stages than horses with restricted access to hay. We found no correlations between the behavioral traits assessed by the owners and the horses’ success in the test. Horses that engaged in many distraction behaviors were more successful than horses that exhibited only few of these behaviors during the waiting time.

### Individual variation in self-control

We observed great individual variation in self-control abilities amongst the horses. Some horses did not manage to pass the 2 s delay, whilst others successfully waited for 60 s. Older horses tended to reach higher delays in Experiment 1 but this effect was not replicated in Experiment 2. Also, sex did not explain individual differences; however, it needs to be noted that no horses below 3.5 years and no stallions were included in the study. The horses’ housing conditions (group-living vs. individual boxes) did not account for the observed individual differences in self-control; however, the hay feeding regime was related to individual performance. In both experiments, we found that horses with permanent access to hay (*ad libitum* feeding) reached or tended to reach higher delay stages compared to horses with only restricted access to hay. While satiation during the course of sessions did not account for success, this effect might be due to two factors, on the one hand, unlimited access to hay might make horses generally more satiated, which in turn can facilitate self-control. On the other hand, general food availability might affect self-control abilities. If food is constantly available without any shortages (as in horses fed hay *ad libitum*), it might be valued differently and more risky foraging decisions for delayed options could be made. For example, honey bees show less self-control, if they are hungry (Mayack and Naug, 2015) and experiences of food shortage reduces self-control in children (Jackson et al., 2018). During food-shortages, immediate energy intake rather than waiting, and thus, increasing the risk of losing all of the available food, is likely more adaptive. While one study found no effect of satiation on self-control abilities in capuchins (De Petrillo et al., 2015), more studies investigating the effects of food availability and resulting relative food values on self-control are warranted. These results open up numerous novel research questions pertaining to the influence of food availability (i.e., hay feeding regime) on general learning capacities, cognitive performance, and emotional states in horses.

Horses that engaged in certain behaviors while waiting for the delayed reward were more successful. In particular, increasing the distance to the LVR, looking away, repetitive horizontal or vertical head movements, pawing, empty chewing, and nibbling of box or body, were related to a better performance in the test. All these behavioral patterns are related to directing the attention away from the available LVR. To tease apart whether these behaviors are indeed a way to divert the attention away from the food reward or rather only represent individual differences in general activity or frustration, one would need to implement a control condition, in which food is present but inaccessible (similar to Evans and Beran, 2007). Interestingly, these behavioral patterns seem to be very similar across animal species from parrots (e.g., Auersperg et al., 2013; Brucks et al., 2021), to canids (Range et al., 2020), cephalopods (Schnell et al., 2021), chimpanzees (Evans and Beran, 2007), and humans (Steelandt et al., 2012). We also observed reward-directed behaviors in horses (i.e., sniffing LVR and pushing LVR away); however, since these behaviors were recorded only very infrequently, we were not able to analyze them. Similar reward-directed behaviors have so far only been reported in chimpanzees (Evans and Beran, 2007), children (Steelandt et al., 2012) and in a gray parrot (Koepke et al., 2015) and were not consistently related to success.

Furthermore, we found that the horses exhibited non-random giving up times, especially during higher delay stages and more frequently in the quality condition compared to the quantity condition. Deciding whether a delayed reward is worth waiting should be made in the beginning of a trial to reduce the amount of effort invested into a reward that is temporally discounted by a delay. Especially, during higher delays it pays to make such waiting decisions early on in a trial. Similarly, also dogs (Leonardi et al., 2012; Brucks et al., 2017b), parrots (Auersperg et al., 2013; Schwing et al., 2017; Brucks et al., 2021), and primates (Dufour et al., 2007; Pelé et al., 2011) give up waiting earlier than expected during higher delay stages. Whether giving up times are purely explained by decisions about anticipated time or rather also by frustration about not receiving the delayed option, however, is difficult to infer.

### Simplified delay of gratification paradigm and effects of test procedures on performance

To make the delay of gratification test more easily implementable in an applied setting, we simplified the test procedure in a way that no additional equipment is needed to conduct the test. Furthermore, since horses were not required to directly exchange food items but rather only refrain from consuming them, we could reduce the training to a minimum. Horses are very sensitive to various human social cues (e.g., Clever Hans; Pfungst, 1911; Proops et al., 2010) and since our simplified test design involved direct interactions with an experimenter we aimed at testing whether one of the potential cues emitted by the experimenter, namely the gaze, affected horses’ performance in the test. For example, dogs refrain from taking food, if a human is directly gazing at them but are less inhibited, if the human closes the eyes (Call et al., 2003). Consequently, also horses might experience social inhibition when the experimenter directly gazes at them. We found that horses reached equally high delay stages independent of whether the experimenter’s eyes were visible (i.e., directly gazing at the horse) or invisible (i.e., wearing sunglasses); accordingly, the presence of the experimenter’s eyes did not induce social inhibition in horses. It needs to be noted that the experimenter could have provided also other subtle behavioral cues to the horses, such as changes in body tension or posture. To rule out effects of such subtle behavior, however, one would need to conduct the test in the absence of an experimenter, for example in an automated setting (e.g., Evans and Beran, 2007) or at hide the experimenter behind a barrier (e.g., Brucks et al., 2017b).

While the results from Experiment 1 showed that the simplified delay of gratification was feasible in a farm setting and resulted in enormous individual variation in performance, we wanted to ensure that the inclusion of multiple experimenters and the rather relaxed success criteria did not affect the horses’ self-control abilities in our simplified test. Consequently, we refined the procedures in Experiment 2 for making the results more comparable to other studies.

Indeed, a comparison between the horses’ performance in Experiment 1 and Experiment 2, revealed differences in the maximally tolerated delay [median exp. 1: 40 s, exp. 2 (quality): 10 s]. Even though individual variation is likely one explanation for the observed differences in self-control abilities between the two study populations, small modifications in the test procedure might also account for it. Firstly, only one female experimenter conducted all tests; thus, reducing the individual variation in the experimenter’s behavior. Secondly, the test environment was more controlled as all horses were tested in the same box with a barrier adjusted to their height. And thirdly, we adjusted the criteria for success to reduce the occurrence of horses passing a delay stage by chance as horses were required to show a stable performance in consecutive sessions instead of passing the criteria only once. To avoid overtraining the horses due to the stricter criterion for success, we reduced the number of delay stages (i.e., fewer incremental steps) in Experiment 2. Furthermore, different reward types were used as LVR in Experiment 1 (hay) and Experiment 2 (lucerne), which could have affected horses’ ability to wait. Indeed, horses needed fewer sessions to reach the criterion in the food preference test in Experiment 1 (mean: 1.18 sessions, range: 1–3) compared to Experiment 2 (mean: 2.39 sessions; range: 1–6); thus, indicating that lucerne was potentially valued higher compared to hay as LVR. These differences in self-control performance of horses in the two experiments indicates that rather small changes in procedure can greatly affect the outcome of studies. Furthermore, control conditions should be implemented to rule out that the horses rely on avoidance learning (i.e., always avoid reward on one side) instead of having a complete understanding of the task’s contingencies. For example, the sides of rewards (left/right) could be randomly switched instead of keeping fixed sides, the order of rewards could be reversed (i.e., HVR first and LVR after delay), or both rewards could be of low quality. But, also the criteria for success, and the experimental paradigms (e.g., standard exchange task and rotating tray task) might affect individual self-control abilities. Certainly, the development of simpler experimental procedures opens up the possibility to collect data on a larger scale (e.g., using a citizen science approach), however, procedural caveats need to be considered carefully when designing such studies. Future studies need to investigate how such procedural differences affect individual self-control abilities.

### Role of self-control in other behaviors

The horse owners’ predictions about their horses’ self-control abilities were not correlated with the actual performance in the test. This is interesting as past research has shown that owners are e.g., able to correctly predict their horses’ reaction in behavioral tests (e.g., Ijichi et al., 2013); however, these behavioral tests did not involve any food rewards. Considering that most horse owners rely on negative reinforcement to train their horses instead of positive reinforcement using food rewards (McLean and Christensen, 2017), they might have only limited experience regarding their horses’ behavior in the context of food. Self-control as assessed in the present study likely is linked very closely to food motivation (as also supported by the influence of the horses’ hay feeding regimen, though contradicted by the missing link to owners’ assessment of their horse’s food motivation), and this is a different motivational axis compared to the motivations that need to be inhibited when, e.g., refraining from showing fear reactions to frightening stimuli or to show other strongly motivated behaviors in a training setting. Additionally, as suggested by McLean and Christensen (2017), training success may be affected by factors such as arousal, affective and attachment states. For example, arousal levels in the present training setting may have been different from those typically encountered for the participating horses in their regular training sessions with their owners. Generally, owners’ assessment of their pets’ behavioral tendencies should always be treated cautiously as pet owners might not be able to accurately predict their animals’ behavior or might define behaviors differently compared to those assessed in experimental studies.

Individual self-control abilities were additionally not correlated with other behavioral traits that were rated by the owners, such as trainability, patience, food motivation, and reactions in stressful situations. In light of the findings that self-control abilities are linked to general intelligence (Moffitt et al., 2011; Beran and Hopkins, 2018), including learning performance (Schnell et al., 2021), we would have expected to observe better self-control in horses that were rated as more trainable and patient; however, this lack of a correlation between trainability and self-control might be due to two aspects. On the one hand, we did not test the horses’ learning capacity or trainability but rather relied on the owners’ assessment, and, on the other hand, it needs to established whether self-control is actually consistent in situations involving food (i.e., delay of gratification paradigm) and situations outside of the food context (i.e., training or handling).

Further investigations into the links between inhibitory control and trainability, but also general coping capacities, are warranted. Individual differences in inhibitory control abilities, and self-control in particular, might be responsible for differences in coping abilities and behavioral flexibility in captive animals. Considering that in humans, self-control can be improved by training (Murray et al., 2016; but see Friese et al., 2017), similar training regimes might be implemented in horse training to improve human-animal interactions and ultimately horse welfare.

### Horses’ self-control abilities in a comparative context

Considering that horses’ foraging behavior requires only little self-control as resources are evenly distributed with slowly changing quality, and they face no delays to access the resource, we would have expected horses to show rather poor self-control in such a food-based delay of gratification paradigm. Contrary to our hypothesis, horses exhibited rather good self-control abilities on a group level. In Experiment 1, the horses waited on average for 36.1 s (median: 40 s), while in Experiment 2, the average of the maximally tolerated delay was 13.4 s (median: 10 s) in the quality condition and 15.1 s (median: 10 s) in the quantity condition. In both experiments, a number of horses successfully waited for 60 s, while, for example, dogs waited for up to 15 min (Leonardi et al., 2012; Brucks et al., 2017b), pigs up to 20 s (Zebunke et al., 2018), chicken up to 7 s (Abeyesinghe et al., 2005). Other non-domesticated species, however, tolerated much higher delay times [e.g., long-tailed macaques: 21 min (Pelé et al., 2010), cleaner wrasse: 480 s (Aellen et al., 2021), ravens: 640 s (Hillemann et al., 2014), and cuttlefish: 130 s (Schnell et al., 2021)]. Interestingly, horses were more successful in the quantity condition compared to the quality condition. All horses passed the food preference test and training in the quantity condition, while several horses failed to pass these pre-tests in the quality condition. Furthermore, starting with the quantity condition subsequently facilitated success in the quality condition but not vice versa. Previous research in other species has shown that many species are more willing to wait for rewards of better quality than quantity (e.g., cockatoos: Auersperg et al., 2013; corvids: Hillemann et al., 2014; dogs: Brucks et al., 2017b; pigs: Zebunke et al., 2018; and children: Miller et al., 2019b). Cleaner wrasse, which regularly encounter quantitative but only rarely qualitative differences in resources in a natural context, tolerated higher delays if the rewards differed in terms of quantity (Aellen et al., 2021). Accordingly, the ability of horses to wait for quantitative, but less so for qualitative rewards, might be linked to their foraging ecology; under natural conditions resources are more evenly distributed and do not differ as strongly in quality (Devenport et al., 2005) as for species with other foraging styles (e.g., dogs, parrots, and corvids). This aspect of horses’ foraging ecology might make it more adaptive to pay attention to differences in quantity.

Other studies, however, suggest that social complexity is the main driver for the evolution of self-control abilities (Amici et al., 2008; Aellen et al., 2021; but see Schnell et al., 2021). Horses live in complex social environments (Krueger, 2008) and dominance regulates access to limited resources (Ingólfsdóttir and Sigurjónsdóttir, 2008); consequently, self-control in social interactions is certainly important as, for example, subordinate horses need to refrain from accessing limited resources in the presence of dominant horses. Accordingly, our results certainly lend some support for this hypothesis; however, since comparable data of self-control abilities in closely-related species is missing, it is difficult to draw strong conclusions. Equidae with their small variation in foraging ecology but differences in sociality (Linklater, 2000) definitely pose an interesting model taxon to further investigate the role of sociality in the evolution of self-control abilities.

## Conclusion

Horses showed great individual variation in their self-control abilities ranging from 0 to 60 s. This variation is partly explained by food availability (i.e., hay feeding regime) and reward type (quality and quantity), but also engagement in distraction behaviors during the waiting period. Our study provides the first data on self-control abilities in a grazing species; thus, broadening our knowledge about underlying evolutionary forces driving the evolution of self-control across animal species. While we found no link between self-control and behavioral traits of horses outside of the test context, we hope that our study gives rise to further research questions related to horse welfare, such as understanding the role of self-control in coping behaviors and general trainability.

## Data availability statement

The original contributions presented in this study are included in the article/Supplementary Material, further inquiries can be directed to the corresponding author.

## Ethics statement

The animal study was reviewed and approved by Animal Welfare Officer, University of Giessen (approval number: JLU_kTV_6_2022). We adhered to the Guidelines for the Treatment of Animals in Behavioural Research and Teaching (Animal Behaviour, 2020). Written informed consent for participation was not obtained from the owners because some owners conducted the study themselves (i.e., in Experiment 1) and the other owners were verbally informed and gave consent for their horses to participate in the study.

## Author contributions

DB and UKvB designed the study. AH collected the data and scored the videos. DB analyzed the data and wrote the first draft of the manuscript. UB reviewed and edited the manuscript. All authors read and approved the submitted version.

## Conflict of interest

The authors declare that the research was conducted in the absence of any commercial or financial relationships that could be construed as a potential conflict of interest.

## Publisher’s note

All claims expressed in this article are solely those of the authors and do not necessarily represent those of their affiliated organizations, or those of the publisher, the editors and the reviewers. Any product that may be evaluated in this article, or claim that may be made by its manufacturer, is not guaranteed or endorsed by the publisher.

## Supplementary material

SUPPLEMENTARY FILE Model diagnostics (i.e., model stability, tests for odds assumptions in CLMM) and outputs of reduced models.

SUPPLEMENTARY VIDEO Video illustrating the food preference test, training and test procedure.

SUPPLEMENTARY DATA 1 Individual characteristics, housing and hay feeding regime of horses participating in Experiments 1 and 2.

SUPPLEMENTARY DATA 2 Results of Kaplan–Meier analyses of error times for each horse.

SUPPLEMENTARY DATA 3 Overview of questions (translated from German) asked in the questionnaire with scoring options and correlations between owners’ answers.

SUPPLEMENTARY DATA 4 Raw data sets of Experiments 1 and 2.
