# Supplementary material for: Horses wait for more and better rewards in a delay of gratification paradigm
Source: Front Psychol. 2022 Jul 22;13:954472. doi: 10.3389/fpsyg.2022.954472 (PMC9355425; doi:10.3389/fpsyg.2022.954472)
Supplement: SUPPLEMENTARY FILE — Model diagnostics (i.e., model stability, tests for odds assumptions in CLMM) and outputs of reduced models. [file Data_Sheet_1.docx]

**Supplementary Material**

**Horses wait for more and better rewards in a delay of gratification paradigm**
Désirée Brucks1,*, Anna Härterich1 & Uta König von Borstel1
1Animal Husbandry, Behaviour, and Welfare Group, Institute of Animal Breeding and Genetics, University of Giessen, Germany

1. ***Experiment 1***
   1. ***Model diagnostics***

Model assumptions were checked by dichotomising the delay stages as at least 0s, at least 2s, etc. and fitting logistic models with those derived response variables. The resulting model estimates were inspected, revealing minor variation (see Table S1 and Fig. S1); thus, indicating that the proportional odds were not strongly violated.

**Table S1.** Estimates of fixed effects after dichotomising the response. Each row depicts the fixed effects’ estimates of a mixed model after the response had been dichotomised to the elevel possible outcomes (at least 0s, at least 2s, etc.). The estimated coefficients did generally not vary much between the splits (with the exception of the variables order and phase); thus, suggesting that the proportional odds assumption was not strongly violated.

| Phase  eyes.invisible | order | z.age | sexM | feeding_times  rationed | Housing  individual | Phase  eyes.invisible:order |
| --- | --- | --- | --- | --- | --- | --- |
| 2.273 | -14.094 | -0.630 | 1.154 | -1.592 | -0.083 | -1.714 |
| 2.102 | -1.469 | -0.749 | 1.114 | -2.711 | -1.042 | -1.320 |
| -5.087 | -9.111 | -0.229 | 1.882 | -1.656 | -2.171 | 2.528 |
| -7.193 | -9.778 | 0.054 | 1.175 | -1.307 | -1.225 | 3.666 |
| -0.033 | -1.426 | 0.535 | 0.171 | -2.134 | -0.728 | -0.206 |
| -1.027 | -1.054 | 0.667 | 0.023 | -2.217 | -0.093 | 0.232 |
| 0.408 | -0.352 | 0.887 | 0.217 | -1.700 | -0.479 | -0.761 |
| 0.818 | 0.248 | 0.854 | 0.257 | -1.921 | 0.038 | -0.793 |
| 1.772 | 0.053 | 1.174 | 0.946 | -2.589 | 0.473 | -1.088 |
| 5.430 | 1.013 | 3.748 | 2.698 | -7.487 | 3.319 | -3.636 |

**Figure S1**. Model estimates plotted per fixed effect after dichotomising the response (points) and the estimates for the full model (horizontal line).

Collinearity was determined by the Variance Inflation Factor (VIF) for a linear mixed model entering a numeric response and the same predictors as in the full model but excluding the interaction term. We used the function *vif* of the ‘car’ package (version 3.0-12; Fox and Weisberg, 2019) and found limited collinearity between the predictors (range VIF: 1.05-1.23). Furthermore, we assessed model stability by dropping horses one at a time and fitting the model to each subset using a function kindly provided by Roger Mundry. Estimates from the obtained subsets were compared to those obtained by the full data set. The model was of good stability (see Table S2).

**Table S2.** Model stability: original and minimum as well as maximum of model estimates for CLMM obtained when excluding random effects one at a time.

|  | **Original estimate** | **Min. estimate** | **Max. estimate** |
| --- | --- | --- | --- |
| 0\|2 | -6.409 | -7.723 | -5.372 |
| 2\|5 | -5.663 | -6.833 | -4.707 |
| 5\|10 | -5.092 | -6.174 | -4.149 |
| 10\|15 | -4.719 | -5.746 | -3.781 |
| 15\|20 | -3.383 | -4.243 | -2.463 |
| 20\|25 | -2.933 | -3.745 | -2.003 |
| 25\|30 | -2.509 | -3.282 | -1.570 |
| 30\|40 | -2.025 | -2.755 | -1.077 |
| 40\|50 | -1.520 | -2.198 | -0.568 |
| 50\|60 | -1.052 | -1.702 | -0.097 |
| Phase (eyes.invisible) | 1.309 | 0.105 | 2.464 |
| order | -0.443 | -0.833 | 0.045 |
| z.age | 0.846 | 0.609 | 1.297 |
| Sex (male) | 0.648 | 0.369 | 1.199 |
| Feeding (rationed) | -2.239 | -2.777 | -1.915 |
| Housing (individual) | 0.094 | -0.441 | 0.509 |
| Phase x order | -1.006 | -1.830 | -0.178 |

**Table S3.** Effects on maximum delay duration based on CLMM excluding the interaction between phase x order (reduced model)

| **Term** | **Estimate** | **SE** | **Chisq** | **df** | **P-value** |
| --- | --- | --- | --- | --- | --- |
| 0\|2 | -7.145 | 1.505 |  |  | <0.001 |
| 2\|5 | -6.403 | 1.430 |  |  | <0.001 |
| 5\|10 | -5.834 | 1.375 |  |  | <0.001 |
| 10\|15 | -5.46 | 1.339 |  |  | <0.001 |
| 15\|20 | -4.123 | 1.209 |  |  | 0.001 |
| 20\|25 | -3.674 | 1.167 |  |  | 0.002 |
| 25\|30 | -3.252 | 1.132 |  |  | 0.004 |
| 30\|40 | -2.770 | 1.101 |  |  | 0.012 |
| 40\|50 | -2.270 | 1.075 |  |  | 0.035 |
| 50\|60 | -1.805 | 1.054 |  |  | 0.087 |
| Phase (eyes invisible) | -0.186 | 0.436 | 0.183 | 1 | 0.669 |
| Order | -0.931 | 0.459 | 4.316 | 1 | 0.038 |
| Age^1^ | 0.933 | 0.455 | 4.491 | 1 | 0.034 |
| Sex (male) | 0.558 | 0.796 | 0.501 | 1 | 0.479 |
| Feeding (rationed) | -2.216 | 0.862 | 7.380 | 1 | 0.007 |
| Housing (individual) | 0.213 | 0.854 | 0.063 | 1 | 0.802 |

1. ***Experiment 2***

**Table S4.** Timeline for test procedure for horses starting with either the quality (QUAL) or quantity (QUAN) condition.

| **QUAL first** | Food preference test quality | Training quality | Test quality | 2-week break | Food preference test quantity | Training quantity | Test quantity |
| --- | --- | --- | --- | --- | --- | --- | --- |
| **QUAN first** | Food preference test quality | Food preference test quantity | Training quantity | Test quantity | 2-week break | Training quality | Test quality |

- 1. ***Statistics overview***

**Table S5.** Overview of models used in Experiment 2

| **Model** | **Type** | **Response variable** | **Predictors** | **Random effects** | **Random slopes** |
| --- | --- | --- | --- | --- | --- |
| CLMM2 | --- | Max. delay (factor) | Age  Sex  Feeding  Phase x Order | Horse ID | --- |
| GLMM1 | poisson | Max. delay (numeric) | Age  Feeding  Phase x Order | Horse ID  Observation ID | --- |
| GLMM2 | binomial | success | Trial number | Horse ID | Trial number |
| GLMM3 | binomial | cbind(choice HVR, choice LVR) | Coping behaviours x delay  Coping behaviours x phase | Session ID nested in Horse ID | Delay  Coping behaviours  Phase x coping behaviours |
| GLMM4 | binomial | cbind(choice HVR, choice LVR) | Age  Sex  Reward type x delay | Session ID nested in Horse ID | Delay  Reward type |

- 1. ***Cumulative linear mixed model (CLMM2)***

**Table S6.** Estimates of fixed effects in CLMM2 after dichotomising the response. Each row depicts the fixed effects’ estimates of a mixed model after the response had been dichotomised to the level of possible outcomes (at least 0s, at least 2s, etc.). The estimated coefficients vary extensively between the splits; thus, indicating that the proportional odds assumption was violated.

| **PhaseQUAN** | **Order** | **z.age** | **sexM** | **feedingRestricted** | **PhaseQUAN:order** |
| --- | --- | --- | --- | --- | --- |
| 36.257 | 19.846 | 0.177 | -43.827 | -8.952 | -10.883 |
| -1.734 | 26.447 | -0.093 | -0.078 | 35.175 | 2.296 |
| 4.325 | 1.578 | -0.218 | -0.374 | -1.252 | -2.072 |
| 5.210 | 2.080 | -0.016 | -0.479 | -1.342 | -2.743 |
| 40.860 | 20.462 | 0.301 | 0.056 | -1.743 | -21.689 |
| 116.909 | 55.854 | 31.778 | 17.587 | -51.750 | -90.441 |
| 227.394 | 118.004 | 78.458 | 41.539 | -93.804 | -180.031 |

- 1. ***Generalised linear mixed model (GLMM1) for maximum delay***

Overdispersion proved to be a problem in the GLMM (dispersion parameter: 2.5), accordingly, we included an observational level random effect (Harrison, 2014), which fixed the overdispersion (dispersion parameter: 0.3). We checked for collinearity and found high Variance Inflation Factors (VIF) for the predictors sex and age (both VIF > 1.34), as well as housing and feeding regime (both VIF > 1.78); consequently, we dropped one of each of the correlated predictors from the final model (i.e. sex and housing). Collinearity was no longer an issue in the reduced model (range VIF: 1.01-1.20).

Model stability was determined by dropping one of the observations at a time and comparison the resulting estimates with the model based on the full data set. The model revealed to be of generally good stability (see Table S7) with an exception for the highest delay stage, which likely resulted from a limited number of observations as only two horses reached this stage.

**Table S7.** Model stability: original and minimum as well as maximum of model estimates in GLMM1 for maximum delay obtained when excluding random effects one at a time.

| **Term** | **Original estimate** | **Min. estimate** | **Max. estimate** |
| --- | --- | --- | --- |
| Intercept | 0.980 | 0.736 | 1.256 |
| PhaseQuan | 2.077 | 1.675 | 2.517 |
| Order | 0.946 | 0.699 | 1.072 |
| z.age | 0.073 | -0.002 | 0.141 |
| Feeding.timesRestricted | -0.431 | -0.560 | -0.316 |
| PhaseQuan:order | -1.202 | -1.449 | -0.896 |
| obs@Intercept@NA | 0.589 | 0.445 | 0.605 |
| ID@Intercept@NA | 0.000 | 0.000 | 0.325 |

- 1. ***Generalised linear mixed model (GLMM2) for success across trials***

To rule out that horses got satiated through the course of a session and were thus more likely to wait during the final trials, we ran an additional binomial model on a trial-by-trial basis. As fixed effect, we set the waiting success (binary: waiting/not waiting) and as predictor, we enter the trial number (numeric: 1-15). A random slope of trial number and horse as random effect were included. Collinearity was not issue (VIF: all = 1) and the random effects were symmetrically distributed. The model proved to be stable. The data set used for this analysis consisted of 8235 observations of 29 horses.

The full-null model comparison revealed no differences between the models (Likelihood Ratio Test: χ^2^ = 1.34, df = 1, p = 0.250). Accordingly, we could not detect an effect of trial number on success (GLMM: -0.008 ± 0.007, z-value = -1.146, p = 0.252).

- 1. ***Generalised linear mixed model (GLMM3) for coping behaviours***

Initially, we included also the correlations between these slopes and the intercepts; however, due to convergence problems and several correlations being close to 1, we had to exclude these correlations again. This led to a small reduction in model fit (logLik: original -1098.98, without correlations: -1133.77). Before we fitted the model, we transformed the variables proportion of coping behaviours and age to a mean of 0 and a standard deviation of 1 to ease model convergence and to facilitate model interpretation.

A model with the 2s delay stage as reference level resulted in negative estimates for the interaction between delay and coping behaviours; thus, rendering the interpretation difficult. This is most likely due to the fact that horses could still succeed in the shorter delays (i.e. 2s and 5s) without performing coping behaviours. As delay times increased, coping behaviours became more pronounced and might be more strongly linked to individual success. Model stability was assessed by dropping grouping factors (i.e. individual horses and individual sessions per horse) one at a time and comparing estimates from these subsets of data with the model based on the full data set. The model proved to be of good stability (see Table S8) and collinearity was not an issue (range VIF: 1.00 – 1.18).

**Table S8.** Model stability: original and minimum as well as maximum of model estimates in GLMM1 for waiting success within sessions obtained when excluding random effects one at a time.

| **Term** | **Original estimate** | **Min. estimate** | **Max. estimate** |
| --- | --- | --- | --- |
| Intercept | -1.206 | -1.376 | -0.808 |
| z.age | -0.107 | -0.215 | 0.072 |
| sexM | -0.596 | -0.764 | -0.347 |
| z.cope | -1.079 | -1.292 | -0.752 |
| PhaseQuan | 0.679 | 0.447 | 0.941 |
| Delay 2s | 2.652 | 2.187 | 3.099 |
| Delay 5s | 1.929 | 1.618 | 2.165 |
| Delay 20s | -3.292 | -4.027 | -2.730 |
| Delay 30s | -6.440 | -8.129 | -5.286 |
| Delay 40s | -10.090 | -12.113 | -8.423 |
| Delay 60s | -14.269 | -18.286 | -11.215 |
| Delay 80s | -22.186 | -42.062 | -18.129 |
| z.cope:PhaseQuan | -0.501 | -0.643 | -0.350 |
| z.cope:Delay 2s | 1.577 | 1.255 | 1.947 |
| z.cope:Delay 5s | 1.745 | 1.447 | 2.245 |
| z.cope:Delay 20s | 0.079 | -0.318 | 0.310 |
| z.cope:Delay 30s | -1.140 | -2.029 | -0.157 |
| z.cope:Delay 40s | -1.988 | -4.468 | 0.087 |
| z.cope:Delay 60s | 0.860 | -7.846 | 4.298 |
| z.cope:Delay 80s | 0.107 | -7.619 | 15.086 |
| sessionID@(Intercept)@NA | 0.855 | 0.507 | 1.301 |
| sessionID@z.cope@NA | 1.196 | 0.894 | 1.579 |
| sessionID@Delay@NA | 0.139 | 0.099 | 0.229 |
| sessionID@(Intercept)@z.cope | 0.035 | -0.629 | 0.839 |
| sessionID@(Intercept)@Delay | -0.196 | -0.750 | 0.295 |
| sessionID@z.cope@Delay | -0.713 | -0.877 | -0.666 |
| ID@Intercept@NA | 0.925 | 0.336 | 1.121 |
| ID@Delay@NA | 0.099 | 0.000 | 0.121 |
| ID@Phase.Quan@NA | 1.415 | 1.061 | 1.754 |
| ID@z.cope@NA | 0.000 | 0.000 | 0.830 |
| ID@Phase.Quan:z.cope@NA | 1.501 | 1.172 | 1.834 |

**Table S9.** Effects of coping behaviours on waiting success excluding the interaction between condition and coping (reduced model).

| **Term** | **Estimate** | **SE** | **lower CI** | **upper CI** | **z-value** | **P -value** |
| --- | --- | --- | --- | --- | --- | --- |
| Intercept | -0.708 | 0.640 | -1.818 | 0.557 | -1.107 | 0.268 |
| Age^1^ | 0.131 | 0.390 | -0.662 | 0.915 | 0.335 | 0.738 |
| Sex (M) | 0.049 | 0.776 | -1.371 | 1.483 | 0.063 | 0.950 |
| Cope behav^1^ | 1.216 | 0.272 | 0.669 | 1.831 | ^2^ | ^2^ |
| Delay 2s | 2.368 | 0.290 | 1.833 | 2.879 | ^2^ | ^2^ |
| Delay 5s | 1.464 | 0.195 | 1.064 | 1.805 | ^2^ | ^2^ |
| Delay 20s | -4.173 | 0.352 | -4.859 | -3.551 | ^2^ | ^2^ |
| Delay 30s | -7.854 | 0.719 | -9.456 | -6.553 | ^2^ | ^2^ |
| Delay 40s | -12.507 | 1.363 | -13.85 | -10.849 | ^2^ | ^2^ |
| Delay 60s | -15.604 | 3.441 | -16.816 | -14.269 | ^2^ | ^2^ |
| Delay 80s | -26.674 | 4.477 | -27.569 | -25.71 | ^2^ | ^2^ |
| Phase (Quan) | 0.682 | 0.381 | -0.058 | 1.493 | 1.791 | 0.073 |
| Cope:Delay 2s | -0.007 | 0.212 | -0.525 | 0.530 | -0.031 | 0.975 |
| Cope:Delay 5s | 0.241 | 0.173 | -0.188 | 0.627 | 1.399 | 0.162 |
| Cope:Delay 20s | 0.543 | 0.184 | 0.064 | 1.056 | 2.945 | 0.003 |
| Cope:Delay 30s | 0.840 | 0.242 | 0.041 | 1.706 | 3.476 | 0.001 |
| Cope:Delay 40s | 1.324 | 0.463 | 0.007 | 2.42 | 2.863 | 0.004 |
| Cope:Delay 60s | 0.270 | 1.176 | -1.042 | 1.543 | 0.229 | 0.819 |
| Cope:Delay 80s | 1.854 | 1.319 | 0.678 | 3.500 | 1.406 | 0.160 |

^1^variables were scaled to a mean of 0 and a standard deviation of one. Original variables: age = 197.06 ± 77.74 months; proportion of coping behaviours per test duration = 0.396 ± 0.381

^2^not depicted due to limited interpretability

^3^Likelihood ratio test for coping x delay: Chisq = 78.154, df = 7, p < 0.001

**Figure S2.** Effects of coping behaviours on the number of successfully waited trials (= choice for high-value reward (HVR)) plotted separately for each delay stage (2s – 80s; both conditions combined). Black points indicate individual data points. The blue line is a regression line based on a simplified linear regression (choice HVR ~ proportion coping behaviours) fitted for each delay stage (using the *geom_smooth* function within the ggplot2 package).

As before we assessed model stability by dropping individual horses as well as sessions one at a time and comparing the derived estimates with those from the full model. Model stability was good (see Table S8) and collinearity was not an issue (range VIFs: 1.00 – 1.12).

- 1. **Generalised Linear Mixed Model (GLMM4) for reward types**

**Table S10.** Model stability: original and minimum as well as maximum of model estimates in GLMM for waiting success within sessions in the quantity condition obtained when excluding random effects one at a time.

| **Term** | **Original estimate** | **Min. estimate** | **Max. estimate** |
| --- | --- | --- | --- |
| Intercept | 1.255 | 1.165 | 1.500 |
| z.age | 0.011 | -0.173 | 0.108 |
| sexM | 0.306 | 0.032 | 0.459 |
| typeLVR | 0.109 | -0.017 | 0.283 |
| Delay 5s | -0.546 | -0.651 | -0.24 |
| Delay 10s | -2.003 | -2.191 | -1.327 |
| Delay 20s | -6.176 | -6.647 | -4.685 |
| Delay 30s | -10.917 | -11.858 | -8.515 |
| Delay 40s | -16.317 | -17.852 | -12.971 |
| Delay 60s | -22.522 | -24.476 | -17.417 |
| Delay 80s | -33.092 | -45.576 | -26.118 |
| typeLVR:delay5 | -0.026 | -0.210 | 0.089 |
| typeLVR:delay10 | -0.123 | -0.253 | -0.044 |
| typeLVR:delay20 | -0.330 | -0.466 | -0.223 |
| typeLVR:delay30 | -0.139 | -0.389 | 0.127 |
| typeLVR:delay40 | -0.065 | -1.790 | 0.404 |
| typeLVR:delay60 | -0.280 | -0.929 | 0.589 |
| typeLVR:delay80 | 1.664 | 1.233 | 14.641 |
| sessionID@Intercept@NA | 1.020 | 0.002 | 1.104 |
| sessionID@Delay@NA | 0.167 | 0.109 | 0.194 |
| sessionID@typeHVR@NA | 0.000 | 0.000 | 1.037 |
| sessionID@typeLVR@NA | 0.569 | 0.538 | 1.219 |
| sessionID@typeHVR@typeLVR | NaN | -0.993 | 1.000 |
| ID@Intercept@NA | 0.000 | 0.000 | 0.083 |
| ID@Delay@NA | 0.112 | 0.090 | 0.140 |
| ID@typeHVR@NA | 0.849 | 0.552 | 0.951 |
| ID@typeLVR@NA | 0.025 | 0.000 | 0.193 |
| ID@typeHVR@typeLVR | 1.000 | -1.000 | 1.000 |

**Table S11.** Effects of reward type on waiting success in the quantity condition excluding the interaction between reward type and delay (reduced model).

| **Term** | **Estimate** | **SE** | **lower CI** | **upper CI** | **z-value** | **P -value** |
| --- | --- | --- | --- | --- | --- | --- |
| Intercept | 1.336 | 0.341 | 0.653 | 1.967 | 3.920 | <0.001 |
| Age^1^ | 0.026 | 0.209 | -0.350 | 0.432 | 0.123 | 0.902 |
| Sex (M) | 0.302 | 0.382 | -0.407 | 1.079 | 0.792 | 0.428 |
| Type (LVR) | -0.035 | 0.202 | -0.398 | 0.367 | -0.174 | 0.862 |
| Delay 5s | -0.556 | 0.186 | -0.888 | -0.253 | -2.985 | 0.003 |
| Delay 10s | -2.051 | 0.330 | -2.552 | -1.618 | -6.214 | <0.001 |
| Delay 20s | -6.310 | 0.696 | -7.511 | -5.411 | -9.072 | <0.001 |
| Delay 30s | -10.950 | 1.082 | -13.262 | -9.351 | -10.121 | <0.001 |
| Delay 40s | -16.308 | 1.487 | -18.989 | -13.281 | -10.965 | <0.001 |
| Delay 60s | -22.624 | 2.337 | -24.224 | -21.683 | -9.681 | <0.001 |
| Delay 80s | -31.901 | 3.158 | -33.132 | -29.944 | -10.102 | <0.001 |

^1^variable was scaled to a mean of 0 and a standard deviation of one. Original variable: age = 197.06 ± 77.74 months

**References**

Fox, J. and Weisberg, S. (2019) *An R Companion to Applied Regression*. 3rd edn. Sage: Thousand Oaks. Available at: https://socialsciences.mcmaster.ca/jfox/Books/Companion/.

Harrison, X. A. (2014) ‘Using observation-level random effects to model overdispersion in count data in ecology and evolution’, *PeerJ*, 2014(1). doi: 10.7717/peerj.616.
